# Functional or Psychogenic Movement Disorders: An Endless Enigmatic Tale

**DOI:** 10.3389/fneur.2015.00037

**Published:** 2015-02-27

**Authors:** Carlo Dallocchio, Antonio Marangi, Michele Tinazzi

**Affiliations:** ^1^Division of Neurology, Ospedale Civile, Azienda Ospedaliera Della Provincia Di Pavia, Voghera, Italy; ^2^Section of Neurology, Department of Neurological and Movement Sciences, University Hospital of Verona, Verona, Italy

**Keywords:** functional neurological symptom disorder, psychogenic movement disorders, conversion disorder, somatoform disorder, psychosomatic symptoms, psychological therapy, physical activity

Functional/psychogenic movement disorders (F/PMDs) are a valuable model for all medically unexplained symptoms and raise arduous challenges for diagnosis and treatment indicating our restricted understanding of the true pathogenesis that causes them.

A multiplicity of terms, such as “conversion,” “somatization disorder,” “psychosomatic,” “neuropsychiatric,” “dissociative motor disorders,” and so on, have been applied to describe neurological symptoms that cannot be attributed to any known organic disease (1).

In recent years, there has been a great debate about the use of the terms “psychogenic” and “functional,” which is far from being solved.

The term “psychogenic” is common and classically used in the movement disorder literature (2), and it refers to a presumed causal relation between psycho(patho)logical factors and the generation of abnormal movements. The role of psychopathological triggers still remains poorly understood; however, it has been shown that patients with PMDs frequently present a higher rate of major emotionally stressful or traumatic life events when compared to healthy volunteers; at the same time, the evaluation of similar parameters in patients with hand dystonia reveals very few differences from patients with FMDs (3). Hence, given that relevant psychological factors may not be demonstrable at the time of diagnosis, in the recently published Diagnostic and Statistical Manual of Mental Disorders (DSM-5), the requirement for the demonstration of the “psychogenicity” of a conversion disorder (or “functional neurological symptom disorder”) is not necessary, while it has been highlighted the importance of an accurate neurological examination, in order to demonstrate the presence of positive diagnostic physical symptoms and signs typical of PMDs, which are not typically seen in other movement disorders (4). Moreover, it should be emphasized that the term “psychogenic” turns out to be linguistically faulty, implying that the movement disorder has “given birth” to the psychiatric problem. Thus, in the DSM-5, F/PMDs are included under the broader category of “functional neurological symptom disorder”; moreover, the umbrella category of “somatoform disorders” disappeared to emphasize more desirable clarity and to point up the prominence of somatic symptoms that cause distress in these disorders (4).

Hence, some authors argue that the term “functional” better reflects the state of the evidence regarding the pathophysiology of psychogenic disorders, and its use is encouraged (1, 5–8), as it could favor acceptance at the time of diagnostic debriefing. Moreover, this term is freer from etiological assumptions, which are poorly understood, and does not reinforce the dualistic thinking regarding the relation between mind and brain (1). While some authors assert that subjects who present with these disorders commonly perceive themselves as “dysfunctional” rather than “functional” (9), it seems reasonable to prefer the use of this term in clinical practice, because it provides the undeniable advantage of avoiding the reference to etiological theories, which are not yet fully demonstrated, making the diagnosis more acceptable to patients.

By the late nineteenth century, psychoanalytic theory ruled medical reasoning about these symptoms. Originally referring to these disorders as hysteria, neuropsychiatrists began illustrating the various clinical phenomenological aspects of such disorders. Charcot proposed that hysteria was congenitally derived, and that lesions responsible for this condition might ultimately be found somewhere in the brain. Today, we know that neuroimaging studies have shown an abnormal network of neuronal activation in brain function in patients with PMDs (10). Regarding therapy, in that era hypnosis was used as a powerful tool for demonstrating how, on occasion, subconscious motivations could generate a variety of disabilities resembling those seen in the context of bona fide neurologic disease; hence, paralysis, tremors, convulsions, and sensory alterations were identified as sometimes being due to hysteria. Subsequently, different etiologies of dystonia, tremor, myoclonus, and other movement disorders were recognized.

Emerging neurobiological evidence, neurophysiological findings, and improved neuroimaging have provided significant insights about the psychogenicity of the diagnosis; nevertheless, it remains unknown whether the alterations reflect etiological mechanisms (10). F/PMDs are generally also associated with emotional and functional disturbances (11), but functional imaging data show that subjects with F/PMDs have sensory deficits and impairment of perceived voluntariness and volition, indicating that F/PMDs are not manifestations of intention (12). Moreover, how emotional stressors originate an alteration in the dynamic integration between awareness of agency and instant sensation, and the control of bodily function still remain to be clarified (13).

More in-depth interaction of interests between neurosciences and cognitive psychology seems to demonstrate a very close connection between cerebral events and mental experiences. These results indicate more than just a limitation to the concept of the mind as a separate metaphysical entity that operates independently from the brain, and seem to recognize the unconscious mind as the center of brain activity that controls our behavior and determines our emotional desires and other mental inclinations (14). As such, the mind does not intervene by changing the universal rules that neural pathway functioning is based on, but exercises a control of these pathways (15). There may be a pathologic unconscious influence on movement production associated with a disconnection between movement production and sense of volition (16). Besides, earliest affective or stress-related factors, neuropsychological and psychosocial processes, perhaps involved primitive reflexive mechanisms of protection and alertness that are not fully independent of conscious control (13).

Despite the important progression in neuroscience knowledge in recent years, patients without any evidence for an underlying organic disorder remain frequent and puzzling in clinical practice.

Unlike other definitions or programs in the medical field, terms like “psychogenic” or mainly “psychosomatic,” result in a division between biological and psychological processes, and moreover, are not very useful because they are too generic, excessively broad in scope, and can determine unfavorable tangible consequences. The psyche is often considered a somewhat mysterious “structure” of the individual to add to the other known “structures.” So initially, these terms that refer to the mind are formulated in an ambiguous manner and seem to refer to physical disturbances with no organic causes and thus of a psychological origin, causing them to be mistaken for “psychogenetic” disorders or even “imaginary” and this is not just in the mass media but by neurologists, psychiatrists, and psychologists as well (17). This attitude runs the risk of creating a type of doctor shopping behavior in patients who feel rejected by medicine and not accepted in relationships with physicians, leading to an abnormal behavior constantly searching for check-ups, exams, medicines, etc. Another possible consequence is defensive medicine by physicians due to a fear of being accused of malpractice and which leads to abnormal prescribing conduct.

On the other hand, even managing to explain to patients that they have a “functional” or “non-organic” disorder is not always easy. We could minutely argue in favor of the use of others terms as “primary,” “distinctive,” “interactional,” etc., therefore trying to avoid the psyche/body dualism, but the main objective is not creating an ambiguous relationship between patients who *live* inside the complexity of their bodies and physicians who *observe* the unhealthy body as a three-dimensional object, which can be measured with analyses and examinations (18). From a clinical viewpoint, the divergence in the physician–patient relationship is clear when listening to the terms used by patients and those used by physicians to describe the same symptoms (19). Speaking about two different aspects of the body should be avoided, they are complementary to each other but do not have a common language. But it is even more important to reflect on the fact that a functional symptom, and particularly an “excessive” movement disorder, is part of direct body language, i.e., one of the most instinctive and primordial expressions of human communication, and is the result of that inseparable whole, which we are.

Psychosomatic diseases do not exist as such but each disease has multiple factors and the patient’s clinical picture needs to the analyzed as accurately as possible on the basis of available instruments and knowledge (20). We know that psychological factors can result in physical illness, but we know very little about how they act. Even when we believe that psychological factors are fundamental in a functional disorder, the possible influence of other neurobiological, sociodemographic, and cultural factors may be equally important, even if we currently do not know their mechanisms (21). Thus, there may be psychosomatic factors in the pain of a Parkinson’s patient and no psychosomatic factor in a patient with an evident functional tremor.

Faced with the heterogeneous clinical presentation of all functional neurological disorders, it is clearly useless from an operating standpoint to have a single diagnostic category, which many patients and therapists are looking for. On the other hand, it is also clear that technical instruments such as scales, questionnaires, neuroimaging, and laboratory investigations are needed to reach a diagnosis. The essential point for those who have to deal with these pathologies is to think and work with different procedures, attempting to carefully consider and differentiate the type and level of association between psychological elements and physical conditions as much as possible (20, 21). Unlike what is commonly believed, patients with functional neurological symptoms want to be heard and receive emotional support, even before receiving diagnostic findings, which explain their symptoms.

There is no consensus even among the experts about the best treatment approach to patients with F/PMDs. Actually, psychological therapy (22) as well as different physical approaches (23, 24) seem to improve symptoms, but well designed prospective studies are needed. Therefore, a common agreement is that treatment begins when the physician has made the diagnosis and mostly depending on the way of explaining F/PMDs to the patient, as well as a very close working relationship between neurologist, consulting psychologist, and frequently physical therapist, is crucial in obtaining symptom remission in many subjects. The objective of effective treatment is not only to provide symptom remission in the short term but also to evaluate the causes that produced the heterogeneous symptomatology and to assess feasible strategies to remove them (25).

The issue about the terminology to use for the diagnosis is unresolved. In any case, whatever term is used it is important to find an explanatory language that engages the patient and gives a scenario within which to understand the disorder. In this regard, a self-rating approach reported that 49% of patients attributed a favorable outcome to a physician’s described treatment (26).

It is very important to hear out the patient with interest, compassion, and empathy (and patience) and reassure him/her early on, for example, emphasizing that this is an “involuntary” condition and is most likely the result of an impairment of neural pathways. Another option is to explain that some of the symptoms are stress-related symptoms, pointing out that stress is a common cause of many physical afflictions.

Another possibility is showing the patient with functional motor symptoms their physical signs (e.g., Hoover sign); if done in the right way, this could be one of the most useful things a neurologist can do for these patients in persuading them of the accuracy of their diagnosis and the potential reversibility of their symptoms (27).

A sincere, supportive, hopeful and, professional manner of approach will allow understand and at the same time have patients understand what the movement disorder means, what its functions are, and why and when it evolved (28). From this point of view, it emerges once again the possible utility of the use of the term “functional” in the communication of the diagnosis.

We are aware that F/PMDs represent “a crisis for neurology” (29) or express a “language crisis” (30). We need to acquire new scientific skills for identifying that union between the mind and body, separated for centuries by dry rationalism, which can finally become an integrated vision where the mind is no longer the entity that directs, structures, and makes sense of the reality it perceives, but is itself intimately linked to this reality and is the most enigmatic part of it.

## Conflict of Interest Statement

The authors declare that the research was conducted in the absence of any commercial or financial relationships that could be construed as a potential conflict of interest.
